# P015: Prevention of re-use of single-use suction catheter decreased the vap incidence density in a resource-limited adult ICU in Egypt

**DOI:** 10.1186/2047-2994-2-S1-P15

**Published:** 2013-06-20

**Authors:** M Enany, M Sherif, AA El Kholy, GL Saeed, E Sobhy

**Affiliations:** 1 Cairo University, Cairo, Egypt

## Introduction

Ventilator-associated pneumonia (VAP) had a major impact on patient morbidity and mortality in our ICU, an adult ICU in a tertiary hospital, in spite of application of ventilator- associated pneumonia prevention (VAP) bundle. Between March 2009 and May 2010 the incidence rate was 17/1,000 device-days

## Objectives

This study aimed to identify and correct unsafe procedures related to care of the ventilated patient

## Methods

Due to limited resources, the suction catheter was found to be reused for the same patient during a working shift (6 hours). This practice was prohibited and the hospital management was convinced to provide adequate regular supplies of single- use suction catheters. A new policy for suctioning was written and implemented

## Results

From June 2010 till December 2012, a new sterile single- use catheter was used for each suction procedure then discarded immediately after single- use. The incidence rate was decreased to 5.6/1,000 device-days by the end of 2012 (67% decrease).

## Conclusion

Re-use of single- use supplies contributes to increasing the rate of healthcare- associated infections in low- resource settings. The infection preventionist should make a business case of infection control and analyze the cost- effectiveness of interventions

## Disclosure of interest

None declared

